# Progress in Surgery

**Published:** 1899-03-18

**Authors:** 


### Progress in Surgery.

### OTOLOGY.

(*Concluded from page 403.*)

**Middle Ear Suppuration**—Treatment: The following are the lines of treatment, modified, of course, according to the severity of the symptoms: The patient should be kept quiet both mentally and physically, given a good saline purge and a light diet. If there be severe pain, febrile symptoms, and considerable hyperæmia, the patient being a healthy adult, he recommends an application of leeches tothe mastoid region. If the case is less severe, or from general ill-health bleeding is contraindicated, warm sedative poultices may be applied externally, or medicated applications instilled. When pain continues, and there is evidence of a collection of pus in the middle-ear, the pus must be evacuated without delay.

In the treatment of the cases coming under the second heading, if there is purulent discharge, De Santi recommends gently syringing with warm boric acid solution, followed by carefully drying and plugging with absorbent wool. If there is much discharge in the early stages, just subsequent to perforation, it may be necessary to do this three or four times daily. Chronic cases, which, unfortunately, are by far the most common, are often extremely difficult and even impossible to cure. The general lines of treatment should be: (*a*) Thorough cleansing and disinfection of the cavities of the middle ear; (*b*) appropriate treatment of the nose, nasopharynx, and throat; (*c*) careful general treatment, especially with regard to such diatheses as tubercle, syphilis, &c. Thorough cleansing and disinfection of the cavities of the middle ear are of the greatest importance, and such treatment is only in accordance with ordinary surgical principles.

Two methods may be employed—the moist and the dry. The former is applicable when there is profuse and fœtid discharge; the latter when syringing causes pain or giddiness, where the discharge is slight and non-fœtid, and in cases of old quiescent perforations (if they are treated at all). He carries out the moist treatment thus: Thorough syringing of the ear with warm boric lotion (100 deg.—102 deg. F.); inflation of the middle ear by Valsalva's or Politzer's method, and careful drying of the parts with absorbent wool wicks twisted on an aural probe; finally, he insufflates a very little finely-powdered boric acid on to the diseased mucous membrane of the tympanum, and lightly fills the outer half of the external meatus with a plug of salicylic wool. If the discharge be very profuse he orders this treatment to be carried out three or four times a day.

De Santi gives a number of other antiseptic solutions which may be used in a similar manner.

**The Dry Method.**—The lessening of the discharge is an indication to diminish the frequency of the syringing, and to employ dry applications. The ear is cleansed with cotton wool wicks and the diseased parts are insufflated with finely-powdered boric acid. A powder formed of boric acid four parts and iodoform one part may be substituted for the plain boric acid, particularly in tuberculous cases.

Tribromphenol-bismuth is commended by Somers[6] as an antiseptic in chronic suppurative otitis media. The composition of tribromphenol-bismuth is indicated by its name, while its physical properties recommend it for aural use, being odourless, impalpable, and non-irritating. It is greenish-yellow in colour, neutral in reaction, insoluble, and, on account of the high temperature required for its decomposition, can be sterilised without affecting its value. As the drug is slowly decomposed in the ear, it exerts its action for a considerable time, keeping the mucous surfaces under its antiseptic influences for a longer period than any other remedy with which he is familiar. Of great importance is the thorough cleansing of the canal and tympanic cavity of all detritus before any powder is applied, and this is especially so here, as the drug will have little or no influence unless brought in immediate contact with the pus-producing surfaces. Under its use epithelial growth is promoted and cicatrisation occurs at an early period, while its sedative action allays pruritus and consequently allows of more rapid repair of the tissues. The author gives a number of cases illustrating the value of this substance.

**Hæmorrhage from the Ear.**—A remarkable case is reported by Stewart[7] in which he had to ligature the common carotid artery for hæmorrhage from the external ear. On arrival he found a little girl, aged three years, lying on a sofa almost lifeless. Three weeks before she had got thoroughly wet, and a few days afterwards a lump appeared at the angle of the left jaw, with pain on swallowing. On the fourteenth day from the appearance of the swelling in the neck the child complained of intense ear-ache, and a few hours afterwards there was a profuse discharge of foul, greenish pus from the left meatus. Forty-eight hours afterwards an attack of hæmorrhage from the ear occurred, followed by another on the same day, and two more on the day following. The bleeding always ended in faintness. On the next day but one she had another attack of hæmorrhage. He packed the external meatus with gauze and bandaged the head up tightly, but the bleeding recurred. The left common carotid artery was then tied, and the child was quite well within a week. The blood was distinctly venous in character. The only change noticeable after operation, and which persisted, was a flushing of the right side of the face, with profuse sweating on exertion, as compared with pallor on the side operated on.

[6] New York Med. Jour., Dec. 25, 1898. [7] Brit. Med. Jour., Feb. 18, 1899.

### LARYNGOLOGY.

**Laryngeal Neurosis.**—An important point has been cleared up by F. Klemperer.[1] The author's investigations were prompted by Grossman's communication, which stated that the median position of the vocal cord which was hitherto considered the result of abductor paralysis was due to complete paralysis of the recurrent; after division of the recurrent the corresponding vocal cord was in the position of marked adduction. Dogs were used in the experiments. When the exposed recurrent was squeezed with forceps, the vocal cord was seen in the middle line; the same position was observed after section. This is due to nerve irritation on section. By careful preparation of the nerve, with a clean cut through the nerve, the vocal cord was not in the middle line. Where it does occur, the median position is only transitory. After a few minutes in all cases the cord was away from the middle line, in the position, which must be described as the cadaveric, it remained. This position is only marked on division of one recurrent. Double division induces stenosis due to diminution of the intratracheal air pressure. The real position after double division is seen first after tracheotomy. After single division of the recurrent, the paralyzed vocal cord is fixed one to two millimètres from the middle line. The other cord is abducted strongly on inspiration, there is no stenosis. After division of one crico-arytenoideus posticus, he found the corresponding vocal cord near the middle line; the arytenoid did not move outwards, and there was no abduction. After double division of the postici, the condition exactly corresponded to that after double abductor paralysis in the human subject.

**Recurrent Paralysis in Mitral Stenosis.**—In the case of a boy[2] aged seventeen, where complete paralysis of the left vocal cord had been observed during life, the necropsy revealed a universally adherent pericardium, while the left auricle was enormously dilated, and pressed upon the recurrent nerve. A similar condition was observed in a woman aged thirty-four, with rheumatic history, there being complete paralysis of the left vocal cord. Postmortem: A button-hole mitral valve was found, with the left auricle immensely enlarged, squeezing the recurrent laryngeal against the aorta and leading to its degeneration.

**Tuberculosis of the Larynx.**—Ricque[3] calls attention to the rare localisation of tuberculosis in children. He reports a case of a child of four and a half years, who had ulceration of the pharynx, which resulted in progressive destruction of the uvula and of the epiglottis, caseification of the cervical ganglions, and, finally, tuberculous and enteric complications. He refers to twelve cases collected by Siégert,[4] two being in his own practice. Of these twelve the uvula was affected in two cases, the tonsils in five, the epiglottis in six, and the tongue and posterior part of the pharynx in nine. The mucous membrane was affected in only one case. The progress is rapid; the prognosis fatal. The best application, especially where there is false membrane, appears to be lactic acid. The fever is absent or slight at the commencement, engorgement of the cervical ganglion constant, and the absence of the Loeffler bacillus is the only means of preventing confusion with diphtheria.

One of the most important contributions of late years on this subject comes from Jobson Horne,[5] who gave in the course of a demonstration some of his observations based upon a research extending over some years. He dealt first with the pathogenesis of the disease—the portal of infection, the beginning and path of the tuberculous destruction, the origin and formation of the giant cell. The pathogenesis of the disease was studied in larynges which post-mortem presented to the naked eye none of the usual signs of laryngeal tuberculosis, but which had been removed from bodies in which there had been undoubted tuberculosis in other organs. The earliest changes noted in the lymphatics were a proliferation of the parenchyma of the acini and efferent ducts, and the formation of masses of small round cells distending and choking the ducts and obliterating the glands, the adjacent and superficial structures remaining intact. These changes he had noted in the lymphatics situated in the walls of the ventricles, when a careful microscopic examination of the entire larynx had failed to reveal changes in the lymphatics in other parts. The nature of this cell proliferation was next considered by Horne. Is it to be regarded as nothing more than an outcome of a traumatism or of some simple catarrhal process perhaps peculiar to tuberculous subjects? Is it to be attributed to one specific cause, namely, the presence of the tubercle bacillus? Horne, although not prepared altogether to accept or reject the theory that these lymphoid masses are due to a simple catarrhal process, was able to demonstrate the tubercle bacilli in the midst of some of them, and is of the opinion that, once having gained an entry into the lymphatic ducts, they act as an irritant and cause a cell proliferation. The *fons et origo* of the giant cell in tuberculosis and its development from the wall of a lymph space was demonstrated. His series of slides showed first the tubercle bacilli lying amongst the endothelial cells forming the wall of the lymph space; secondly, the fusion of the adjacent and divided cells; and, thirdly, the separation off of this plasmodial mass as a giant cell. Horne referred to the researches on lepra laryngis by Dr. Paul Bergengrün, of Riga, in which he had shown that the so-called "globi" were bacillary thrombi lying in dilated lymphatics, and that the lepra giant cell developed from the lymphatic endothelium. If, then, as would appear to be the case from the foregoing, the tuberculous process commences in the lymphatics, it would be natural to suppose that those parts which are rich in lymphatics would be the more common sites for infiltration and ulceration, while such parts as are without glands would escape ulceration except by continuity. This was clinically verified. The interarytenoid region, the posterior third of the cord, the ventricular band, and the epiglottis, especially the petiolus, are the parts more richly endowed with glands, and these are the parts more commonly ulcerated in laryngeal tuberculosis. The cord, the functionally more important part of the cord—namely, that part lying between the two small cartilaginous bodies—being free from glands, escapes ulceration excepting superficial erosion and by continuity. A proliferation of the sub-epithelial blood vessels takes place side by side with this cell proliferation. With regard to the muscles, he described changes as occurring at an early stage in the fibres, and clinically brought into evidence by an early functional failure which was mainly myopathic.

Whilst clinically investigating these pathological changes it was necessary, inasmuch as only slight departures from the normal were being looked for, to retain a control mental image of a normal larynx. This was done by constantly examining a large number—some hundreds in all were examined—of larynges in healthy subjects, or at least presumably free from tuberculosis. And it was whilst in search of normal larynges that some important aids to the present research were stumbled upon. The larynx had been examined in 359 consecutive cases of pulmonary tuberculosis or cases in which there were reasons for suspecting the disease. The points to which attention was mainly directed were the following: (1) Disturbances of sensation, hypoæsthesia, hyperæsthesia, paræsthesia; (2) colour changes, anæmia, hyperæmia; (3) functional disturbances; (4) impaired movements of vocal cords apart from paralysis; (5) changes in the contour of the larynx due to slight œdema.

[1] Ueber die Stellung der Stimmbänder nach Recurrens und nach Posticusdurschneidung. (Jahresversammlung des Vereins süddeutscher Laryngologen), May, 1898. Jour. of Laryng., Oct., 1898. [2] Med. Times and Register, xxxv., No. 11. [3] Ann. des Mal. de l'orcile, No. 5, Mar. 1898. Jour. of Laryng., Sept., 1898. [4] Jahnsuch für Kinderheillunde, xlv. [5] Brit. Med. Jour., Oct. 22, 1898.

